# Sarcopenia and outcomes after revascularization for peripheral arterial disease: a scoping review

**DOI:** 10.3389/fmed.2026.1916774

**Published:** 2026-09-30

**Authors:** Yajiao Liu, Wei Jin, Tiantian Zhou

**Affiliations:** Department of Nursing, The Second Affiliated Hospital of Zhejiang University School of Medicine, Hangzhou, China

**Keywords:** peripheral artery disease, prognosis, revascularization, sarcopenia, scoping review

## Abstract

**Objectives:**

Sarcopenia, a degenerative skeletal muscle disease associated with aging and chronic diseases, is a recognized risk factor for poor postoperative outcomes in various surgical fields. However, in patients with peripheral artery disease (PAD) undergoing revascularization, systematic evidence on its epidemiology, assessment practices, and prognostic impact is lacking. This scoping review aims to synthesize available evidence to clarify the prevalence, diagnostic methods, and association between sarcopenia and adverse postoperative outcomes in patients with PAD, thereby informing perioperative risk stratification and targeted interventions.

**Methods:**

This study was conducted following the Arksey & O'Malley scoping review framework and the Joanna Briggs Institute (JBI) revised scoping review methodology. Relevant studies were retrieved from PubMed, Embase, Web of Science, Cochrane Library, CNKI, WanFang Data, and VIP Chinese Science and Technology Journal Database from inception to June 30, 2025. Eligible studies were published in Chinese or English and focused on the impact of sarcopenia on the prognosis of patients with PAD. The extracted data were collated, categorized, and summarized.

**Results:**

Twelve studies were included. The prevalence of sarcopenia in patients with PAD ranged from 9.01% to 66.67%. Most existing studies diagnosed sarcopenia solely based on imaging-derived muscle parameters without comprehensive assessment of muscle strength and physical function. Despite these methodological limitations, imaging-evident muscle loss was correlated with elevated postoperative mortality, increased risk of cardiovascular events, and poor limb prognosis in patients with PAD.

**Conclusions:**

Sarcopenia is prevalent among patients with PAD and serves as a potential risk factor for adverse postoperative outcomes. Inconsistent diagnostic criteria and substantial methodological heterogeneity across current studies restrict the generalizability of available evidence. Future efforts are required to establish a standardized, multidimensional assessment system for sarcopenia, and high-quality prospective studies are needed to clarify its perioperative prognostic value.

## 1 Introduction

Peripheral artery disease (PAD) is a vascular disorder caused by atherosclerosis and thromboembolism, leading to intermittent claudication, rest pain, ischemic ulcers, and even limb necrosis (1–3). With over 230 million patients globally, PAD imposes a substantial public health burden owing to its associated cardiovascular events, disability, and mortality (4–6).

Revascularization constitutes the mainstay of treatment for PAD. PAD patients are at a high risk of developing sarcopenia due to advanced age, chronic limb ischemia, persistent inflammation activation, and limited mobility (7–9). Sarcopenia is a syndrome characterized by the progressive loss of skeletal muscle mass, strength, and physical function (10). Sarcopenia impairs physiological reserve and stress tolerance, increasing postoperative complications (11–13), prolonging hospital stays (14–16), and raising mortality (17–19), as confirmed by systematic reviews and real-world studies.

Although the prognostic significance of sarcopenia has been well recognized across multiple disciplines, there is a lack of systematic evidence regarding its epidemiological features and correlations with postoperative survival and limb functional recovery in PAD patients undergoing revascularization. This scoping review specifically focuses on PAD patients undergoing revascularization rather than the broader PAD cohort. Clinically, patients eligible for revascularization generally present with advanced-stage disease, severe limb ischemia, and a higher cardiovascular risk burden. The revascularization procedure further imposes perioperative stress and tissue injury. In this context, sarcopenia not only reflects long-term malnutrition and metabolic abnormalities but also directly compromises patients’ tolerance to surgical trauma, and may be closely associated with postoperative recovery, wound healing, long-term functional prognosis, and major adverse cardiovascular events. Therefore, there is a clear need for targeted evidence synthesis specifically in this revascularized cohort.

However, previous studies have demonstrated inconsistencies in sarcopenia assessment approaches and diagnostic cut-off values, and most are small-sample retrospective analyses, resulting in substantial interstudy heterogeneity and posing methodological barriers to evidence synthesis. Therefore, we aim to systematically synthesize the existing literature via a scoping review to characterize the prevalence and assessment strategies of sarcopenia, as well as its association with major postoperative outcomes among patients with PAD undergoing revascularization. The findings will provide an evidence base for future high-quality clinical studies.

## 2 Materials and methods

### 2.1 Study design

This scoping review was conducted according to the Arksey & O'Malley scoping review framework (20) and the Joanna Briggs Institute (JBI) revised scoping review methodology (21). The findings were reported following the Preferred Reporting Items for Systematic Reviews and Meta-Analysis extension for Scoping Reviews (PRISMA-ScR) checklist (22). Unlike systematic reviews that perform quantitative meta-analyses or narrative reviews that rely on subjective summarization, this scoping review aimed to map the research landscape, analyze methodological variations, and summarize key findings. No quantitative synthesis or risk-of-bias assessment was performed. Ethical approval was not required. A review protocol was not registered, as this scoping review aimed to map existing literature rather than test a specific hypothesis, and registration is not mandatory for scoping reviews according to current JBI guidance.

### 2.2 Research questions

- What is the prevalence and population characteristics of sarcopenia in patients with PAD?
- What are the current assessment methods and diagnostic criteria for sarcopenia?
- What are the impacts of sarcopenia on postoperative outcomes in patients with PAD?

### 2.3 PCC (population, concept, context) framework

- Population: Adult patients with clinically confirmed PAD who underwent revascularization.
- Concept: Sarcopenia was diagnosed according to international standards, including but not limited to the Asian Working Group for Sarcopenia (AWGS) (23) and the European Working Group on Sarcopenia in Older People (EWGSOP) (24). Imaging measurements, such as computed tomography (CT) and magnetic resonance imaging (MRI), are also accepted for sarcopenia diagnosis. We objectively summarized different assessment approaches adopted in primary studies without comparing the advantages and disadvantages of various diagnostic criteria.
- Context: Original studies (cohort, case-control, RCTs) in clinical settings that examined the association between sarcopenia and postoperative outcomes.

### 2.4 Eligibility criteria

Inclusion criteria were as follows: (1) clear PAD diagnosis and revascularization treatment methods received by patients; (2) complete sarcopenia diagnostic data (indicators, methods, thresholds); (3) defined postoperative outcomes (such as complications, mortality, functional recovery).

Exclusion criteria were as follows: (1) studies not published in Chinese or English, full-text unavailable, duplicates; (2) reviews, conference abstracts, policies, guidelines, protocols; (3) no clear sarcopenia diagnostic thresholds or criteria.

### 2.5 Search strategy

Studies were retrieved from PubMed, Embase, Web of Science, Cochrane Library, CNKI, WanFang Data, and VIP Chinese Science and Technology Journal Database. Both free-text terms and subject headings were used. The search period was from database inception to June 30, 2025. Publications released after the search cutoff date are included solely as background reference material and are not incorporated into the literature screening process of this review. To minimize the risk of overlooking gray literature, Google Scholar was searched, and the first 20 pages of results were reviewed according to relevance. The search terms included peripheral arterial disease, arterial occlusive diseases, chronic limb-threatening ischemia, intermittent claudication, critical limb ischemia, sarcopenia, muscular atrophy, and muscle loss. A more detailed search strategy developed using subject terms and free terms is provided in Supplementary Document S1.

### 2.6 Literature screening and data extraction

After removing duplicates using EndNote, two reviewers independently screened literature based on titles and abstracts. A standardized data extraction form was developed and piloted. Two reviewers independently extracted data. Any discrepancies were resolved through discussion until consensus was reached, or by consulting a third reviewer. We designed a data extraction checklist to facilitate data extraction by study members, which is presented in Supplementary Document S2. The extracted data included the author, publication year, country, study population, sample size, study design, sarcopenia assessment method, diagnostic criteria, and impact of sarcopenia on postoperative outcomes. Study findings are presented in tabular format, followed by a narrative summary of the results.

### 2.7 Evidence synthesis strategy

Following the standard procedures of scoping reviews, this study used qualitative narrative synthesis for literature analysis, without conducting quantitative meta-analysis. This review aims to fully map the overall research landscape concerning the impact of sarcopenia on postoperative outcomes in patients with PAD, summarize research characteristics and progress in this field, identify existing limitations, and propose directions for future studies. All data were sorted and organized, and the results are presented in descriptive summary tables. The tables include the authors, country, publication year, sample size, study design, sarcopenia assessment tools, diagnostic criteria, prevalence, and primary outcome indicators of each included study.

## 3 Results

### 3.1 Literature search and included studies

In accordance with scoping review methodology, we did not perform quantitative risk-of-bias assessment but used systematic descriptive synthesis. A total of 2,334 articles were initially retrieved. After removing duplicates, 1,838 articles remained. After screening titles and abstracts, 1,758 articles were excluded. The remaining 80 articles were reviewed in full text, and 68 were excluded (5 with unavailable full text, 52 irrelevant to the topic, 5 without a clear sarcopenia definition, and 6 including non-surgical patients). Finally, 12 studies were included. The literature screening flow diagram is presented in Figure 1.

**Figure 1:**
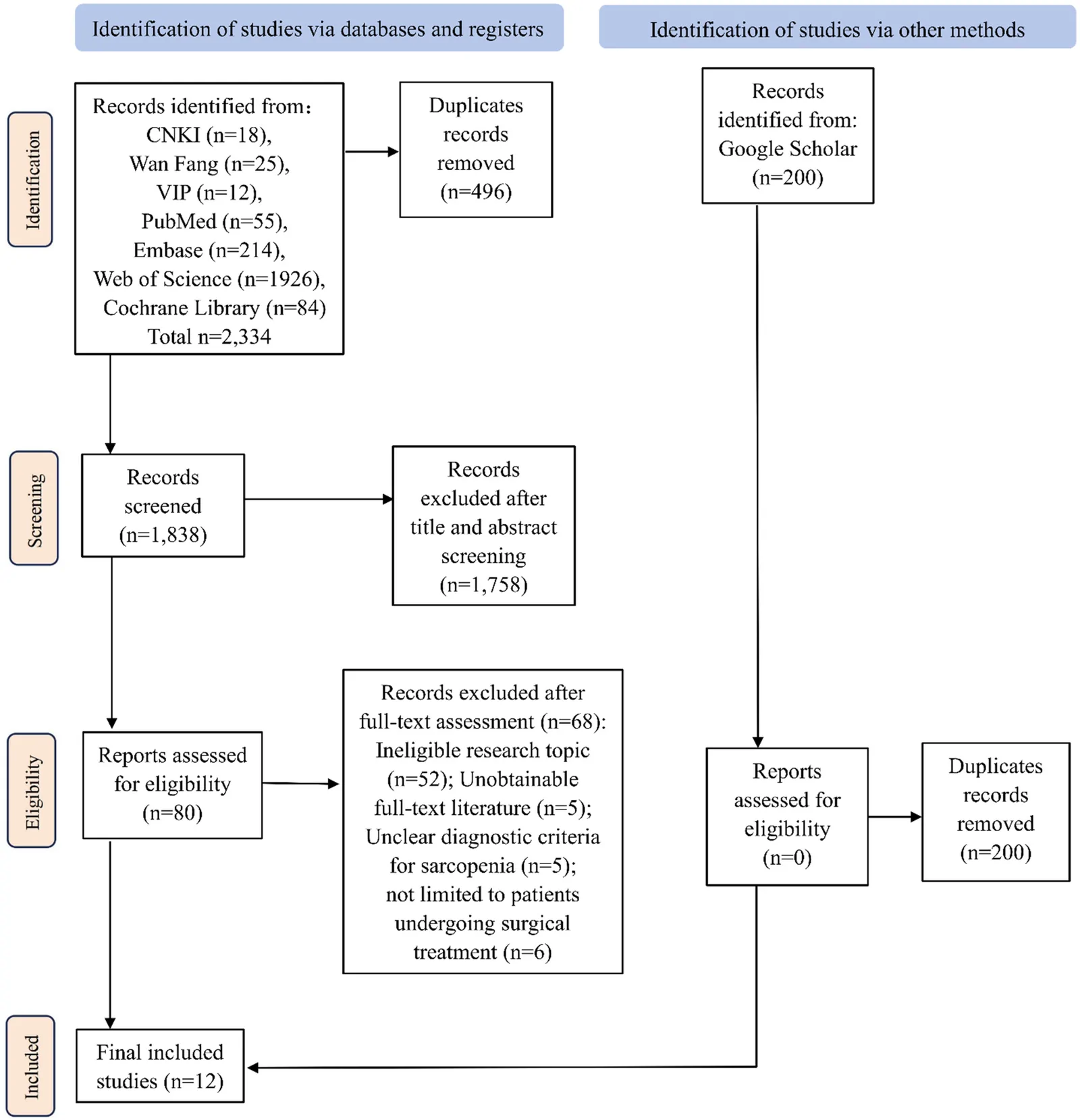
The literature screening flow diagram.

The 12 included studies were published between 2015 and 2025; six (25–30) were from Japan, three (31–33) were from Turkey, one (34) was from the United Kingdom, one (35) was from China, and one (36) was from Finland. The sample sizes ranged from 64 to 899. Only one (30) study was multicenter; the rest were single-center studies. Information on these articles is shown in Table 1. Information regarding intervention modalities, PAD severity, follow-up duration, and multivariable analysis is presented separately in Supplementary Document S3.

**Table 1:** Basic characteristics of the included literature (*n* = 12).

Study (year)	Country	Sample size	Sarcopenia assessment tool	Measurement level	Muscle indicator	Cut-off	Prevalence (%)	Survival Prognosis	limb prognosis	CVD events
Söderlund et al. (2024) (36)	Finland	899	MRI	L4	PMA	Lowest quartile	9.01	Poorer	NR	Increased
Morisaki et al. (2020) (25)	Japan	117	CT	Mid-thigh	STMA	STMA < 9	29.06	Poorer	NS	NR
Cao et al. (2023) (35)	China	137	CT	L3	SMI	M < 40.8, F < 34.9	40.88	Poorer	NS	NR
Taniguchi 2019 (26)	Japan	75	CT	L3	LPI	Lower two-thirds	66.67	Poorer	Poorer	NR
Dağlı et al. (2025) (31)	Turkey	100	CT	L3	SMA	M < 114, F < 89.8	35.00	Poorer	Poorer	NR
Matsubara et al. (2016) (27)	Japan	114	CT	L3	SMA	M < 114, F < 89.8	46.49	Poorer	NR	Increased
Matsubara et al. (2015) (28)	Japan	64	CT	L3	SMA	M < 114, F < 89.8	43.75	Poorer	NR	NR
Selçuk et al. (2022) (32)	Turkey	217	CT	L3	PMI	M < 5.5, F < 4.0	37.79	Poorer	NS	Increased
Guler 2022 (33)	Turkey	190	CT	L3	PMI	M < 3.7, F < 2.5	33.68	Poorer	Poorer	NR
Takino et al. (2024) (29)	Japan	105	AWGS 2019	Multiple	Mass & strength & function	AWGS criteria	31.43	NR	NR	NR
Sivaharan et al. (2021) (34)	UK	116	CT	L3	SMA	M < 114, F < 89.8	31.43	Poorer	Poorer	NR
Shimazoe et al. (2019) (30)	Japan	110	CT	L3	SMA	M < 114, F < 89.8	20.91	Poorer	Poorer	NR

M, male; F, female; NS, no significant association; NR, not reported; CVD, cardiovascular disease; PMA, psoas muscle area; TMA, thigh muscle area; TBA, total body area; STMA, standardized thigh muscle area (TMA/TBA); SMI, skeletal muscle index; LPI, L3 psoas index; SMA, skeletal muscle area; PMI, psoas muscle index; CT, computed tomography; MRI, magnetic resonance imaging; AWGS, Asian Working Group for Sarcopenia.

### 3.2 Prevalence of sarcopenia in PAD patients

Of the 12 included studies, 8 studies (25–28, 30, 32, 33, 35) focused on patients with CLTI, with reported sarcopenia prevalence ranging from 20.91% to 66.67% (of which 6 studies reported rates falling between 33.68% and 66.67%); 2 studies (29, 31) enrolled patients with unspecified-stage PAD, reporting prevalence rates of 31.43% and 35.00%; 1 study (34) included a mixed population of unspecified-stage PAD and CLTI, with a prevalence of 31.43%; and 1 study (36) included a mixed population of intermittent claudication and CLTI, reporting a relatively lower prevalence of 9.01%. In general, patients with sarcopenia were older, had lower body mass index (BMI), and presented with a higher burden of comorbidities.

### 3.3 Assessment methods of sarcopenia in PAD patients

Imaging-based assessment of muscle mass was the predominant diagnostic method in the included studies. CT was the most frequently utilized modality, with the majority of studies measuring the cross-sectional areas of the psoas major and erector spinae muscles at the third lumbar vertebral level (L3). The derived metrics included the skeletal muscle index (SMI) and psoas muscle index (PMI). One study (36) used MRI to measure psoas muscle area at the L4 vertebral level, and one (25) measured mid-thigh muscle area (TMA) using CT. Only one study (29) used the comprehensive criteria of the AWGS 2019, combining muscle mass, strength, and physical function. Consequently, most of the included studies relied solely on muscle mass parameters without concurrent evaluation of muscle strength or physical function.

### 3.4 Impact of sarcopenia on postoperative outcomes in PAD patients

We synthesized evidence regarding survival status, limb conditions, cardiovascular events, and other postoperative outcomes based on extracted data.

Eleven studies (25–28, 30–36) reported the impact of sarcopenia on survival. All studies consistently found that sarcopenia was associated with poorer survival, with lower survival rates and shorter overall survival times.

Eight studies (25, 26, 30–35) reported associations between sarcopenia and limb outcomes. Two studies (31, 34) explicitly defined amputation as major amputation and found a significant association with sarcopenia; two studies (26, 33) reported amputation rates without distinguishing the level of amputation, and one study (30) used amputation-free survival as an endpoint, all reporting significant associations. Additionally, two studies (25, 32) employed limb salvage rate and major amputation rate as endpoints, respectively, and one study (35) did not define amputation separately, all reporting no significant associations.

Three studies (27, 32, 36) reported the relationship between sarcopenia and cardiovascular prognosis, all of which demonstrated that sarcopenia increased the prevalence of cardiovascular events.

One study (29) observed that sarcopenia may lead to poor postoperative functional recovery, manifested as decreased mobility and delayed functional rehabilitation. One study (31) found that sarcopenia was associated with prolonged hospitalization, whereas another study (34) did not confirm this. No significant impact on postoperative complications (25, 31, 34) or vascular patency (26, 34) was observed.

## 4 Discussion

This scoping review included 12 studies and found that sarcopenia prevalence in patients with PAD ranges from 9.01% to 66.67%, which exceeds the 10%–27% reported in community-based studies (37) and the 14.7% in the general Chinese elderly population (38). This higher prevalence may stem from shared pathophysiological mechanisms—chronic inflammation (IL-6, TNF-α), oxidative stress, and insulin resistance—that drive both atherosclerosis and muscle proteolysis via the ubiquitin-proteasome pathway (39). Additionally, ischemic pain reduces physical activity, exacerbating muscle atrophy and creating a vicious cycle of ischemia, sarcopenia, and functional decline (40, 41). Notably, the sarcopenia prevalence reported in this review represents only a specific population of PAD patients undergoing revascularization. In the overall PAD population, a systematic review including a total of 2,362 PAD patients reported an overall sarcopenia prevalence of 34.63% (8); Addison et al. reported a prevalence of 25% in a community-based cohort of elderly male PAD patients (42). These differences in prevalence may be attributable to variations in disease severity across study populations: patients undergoing revascularization are more likely to have advanced-stage disease with a higher proportion of CLTI. Compared with patients receiving conservative management for intermittent claudication or asymptomatic PAD, this group experiences more pronounced ischemic damage, accompanied by greater mobility limitations, malnutrition, frailty, and systemic inflammatory burden. Collectively, these multiple factors contribute to the relatively higher sarcopenia prevalence observed in revascularized PAD cohorts.

Existing observational studies suggest a potential association between sarcopenia and adverse postoperative outcomes such as mortality and cardiovascular events; however, findings remain inconsistent with respect to limb prognosis, length of hospital stay, and postoperative complications. A major methodological limitation of the available evidence is that the vast majority of included studies define sarcopenia solely by measuring skeletal muscle area at a single cross-sectional level via CT or MRI. Importantly, imaging-identified muscle loss only quantifies muscle quantity and cannot be equated with the clinical syndrome of sarcopenia. According to international consensus guidelines, the standardized diagnostic framework for clinical sarcopenia requires evaluation of reduced muscle strength, alongside assessments of muscle mass and physical performance (23, 24). Restricting assessment to cross-sectional muscle area carries a high risk of misclassification: patients with preserved muscle mass but impaired muscle strength and mobility may be incorrectly labelled non-sarcopenic, while individuals with mild muscle loss yet intact physical function may be falsely identified as sarcopenic. This oversimplified, imaging-only operational definition fails to capture the full multidimensional sarcopenia phenotype, may attenuate true prognostic relationships, and represents a key source of inter-study heterogeneity observed across the literature.

In this scoping review, the L3 vertebral level was the most frequently used anatomical site for CT-based muscle mass assessment, and the measured skeletal muscle area has been widely employed to estimate total-body muscle mass. However, this approach essentially provides an estimate of whole-body muscle quantity and cannot directly reflect the actual status of local lower-limb muscles. Tsai et al. (9) demonstrated that the decline in leg muscle area precedes abdominal muscle loss in patients with PAD, and that more severe arterial stenosis correlates with greater lower-limb muscle atrophy, suggesting that local lower-limb muscle indices are more closely aligned with the pathophysiology of PAD. In terms of prognostic performance, Zhao et al. (43) systematically compared muscle indices at different anatomical levels and found that the lower-limb muscle index exhibited superior predictive ability for long-term survival among PAD patients relative to conventional sites such as L3 and L4. Additionally, Lee et al. (44) indicated that reliance solely on the L3 level may underestimate total-body muscle loss, whereas multi-regional combined CT muscle-area assessment can improve the identification of sarcopenia. This is particularly critical for PAD populations, in whom muscle loss presents marked regional heterogeneity. However, at present, there is no unified consensus regarding the optimal measurement site for CT-based sarcopenia assessment in PAD patients. Future studies should systematically compare diverse assessment strategies to establish the optimal protocol for sarcopenia evaluation in this population.

Furthermore, the studies included in this review exhibited substantial methodological limitations. All eligible studies were observational in design, with the majority being single-center, small-sample retrospective investigations. Selection bias is inherently difficult to avoid in such study designs, and key confounding factors were often inadequately adjusted for. Consequently, the available data can only suggest correlational relationships and cannot establish a definitive causal link between sarcopenia and adverse postoperative outcomes. In addition, the lack of uniform diagnostic criteria and the heterogeneity of study populations further limit the generalizability of the current findings.

It is important to recognize that the majority of included studies were derived from cohorts of patients with chronic limb-threatening ischemia (CLTI). CLTI differs substantially from intermittent claudication and asymptomatic PAD with respect to frailty status, risk of malnutrition, amputation rates, and all-cause mortality. Therefore, the conclusions of this review are more applicable to CLTI patients. Future research should evaluate the prognostic value of sarcopenia separately across different PAD clinical subgroups, in order to develop more targeted risk stratification tools for patients at different risk levels.

Despite the aforementioned limitations, imaging-derived sarcopenia-related metrics retain certain clinical utility in specific scenarios. For instance, Kärkkäinen et al. demonstrated that psoas muscle area effectively predicted complications and mortality following complex endovascular aortic repair (45); similarly, Tanaka et al. found that psoas muscle density was associated with long-term postoperative survival and functional recovery in patients with abdominal aortic aneurysm (46). These findings suggest that when comprehensive strength and functional assessments are not feasible, imaging metrics can serve as practical risk stratification tools. Valente et al. identified a major limitation of relying solely on single-slice lean psoas muscle quantification: lean psoas muscle area failed to act as an independent predictor of major morbidity and mortality after thoracoabdominal aortic aneurysm open repair (47), indicating that isolated radiological muscle measurements lack sufficient predictive power without complementary functional, nutritional, and frailty assessments. Therefore, imaging metrics may be used as adjunctive tools, but sarcopenia diagnosed solely by imaging should be interpreted with caution, and a comprehensive evaluation incorporating muscle quantity, muscle quality, physical function, frailty status, and nutritional assessment is warranted.

Based on available research findings, clinicians should prioritize the management of sarcopenia in patients with PAD throughout the perioperative period. For patients with PAD scheduled to undergo revascularization, systematic preoperative screening can facilitate early identification of high-risk individuals, thereby providing a basis for subsequent interventions. In high-risk patients with coexisting sarcopenia, targeted strategies such as nutritional support and exercise prehabilitation may be implemented to mitigate muscle loss and improve postoperative functional recovery. Furthermore, given that older patients with PAD often present with multiple comorbidities and reduced physiological reserve, the establishment of a multidisciplinary collaborative mechanism involving vascular surgery, geriatrics, clinical nutrition, and rehabilitation medicine is particularly warranted. This integrated approach is expected to enable comprehensive perioperative management—from preoperative optimization and intraoperative care to postoperative rehabilitation—thereby improving overall patient prognosis. However, as highlighted in recent clinical practice guidelines, the current lack of standardized assessment protocols has led to insufficient awareness and recognition of sarcopenia among clinicians (48). Therefore, there is an urgent need to develop standardized screening and evaluation pathways specifically tailored to patients with PAD, to increase the clinical detection rate of sarcopenia and optimize perioperative risk management. Future high-quality prospective studies should simultaneously collect data on muscle mass, muscle strength, and physical function within a unified operational framework, so as to enhance diagnostic consistency and the clinical interpretability of findings, ultimately providing more comprehensive, precise, and clinically actionable risk stratification strategies for this patient population.

## 5 Conclusion

This review indicates that sarcopenia is prevalent among patients with PAD and may be associated with increased postoperative mortality, higher cardiovascular event risk, and adverse limb prognosis. However, because most included studies involved CLTI patients, the findings should not be over-generalized to all PAD patients. The evidence is heterogeneous, largely from small, single-center retrospective studies, and most of these studies defined sarcopenia only by muscle mass on imaging, without assessing strength or function. Future prospective multicenter studies using standardized multidimensional assessments are needed to clarify the independent prognostic role of sarcopenia in PAD and to support robust perioperative risk stratification.

## Data Availability

The original contributions presented in the study are included in the article/Supplementary Material, further inquiries can be directed to the corresponding author.
