# Supplementary material for: Sarcopenia and outcomes after revascularization for peripheral arterial disease: a scoping review
Source: Front Med (Lausanne). 2026 Sep 30;13:1916774. doi: 10.3389/fmed.2026.1916774 (PMC13661405; doi:10.3389/fmed.2026.1916774)
Supplement: Supplementary file 1 [file Table1.docx]

## **Supplementary Table 1. Search strategy in PubMed**

| **Database** | **Search strategies** | **Results** |
| --- | --- | --- |
| PubMed | #1 "Peripheral Arterial Disease"[MeSH Terms] OR "peripheral artery disease*"[Title/Abstract] OR "peripheral arterial disease*"[Title/Abstract] OR "arterial occlusive disease*"[Title/Abstract] OR "critical limb ischemia"[Title/Abstract] OR "chronic limb threatening ischemia"[Title/Abstract] OR "intermittent claudication"[Title/Abstract] | 40,817 |
|  | #2 "sarcopenia"[MeSH Terms] OR "Sarcopenias"[Title/Abstract] OR "muscular atrophy"[Title/Abstract] OR "muscle loss"[Title/Abstract] | 27,819 |
|  | #3 #1 AND #2 | 55 |
