# Supplementary material for: Sarcopenia and outcomes after revascularization for peripheral arterial disease: a scoping review
Source: Front Med (Lausanne). 2026 Sep 30;13:1916774. doi: 10.3389/fmed.2026.1916774 (PMC13661405; doi:10.3389/fmed.2026.1916774)
Supplement: Supplementary file 2 [file Table2.docx]

## **Supplementary Table 2. Data extraction table of studies**

| **Item** | **Description** |
| --- | --- |
| **1. General information** | |
| 1.1 Title |  |
| 1.1 First author, year |  |
| 1.2 Country |  |
| 1.3 Study design |  |
| 1.4 Sample size (n) |  |
| **2. Sarcopenia assessment** | |
| 2.1 Assessment tool (e.g.,CT / MRI / other) |  |
| 2.2 Measurement level (e.g., L3, L4, mid-thigh) |  |
| 2.3 Muscle mass indicator (e.g., SMI, PMI, TMA) |  |
| 2.4 Diagnostic criteria / cut-off values |  |
| 2.5 Sarcopenia prevalence (%) |  |
| **3. Postoperative outcomes** | |
| 3.1 Survival |  |
| 3.2 Amputation |  |
| 3.3 Cardiovascular events |  |
| 3.4 Functional recovery / mobility |  |
| 3.5 Hospital stay |  |
| 3.6 Postoperative complications |  |
| 3.7 Vascular patency |  |
| **4. Key conclusion** |  |
