# Supplementary material for: Sarcopenia and outcomes after revascularization for peripheral arterial disease: a scoping review
Source: Front Med (Lausanne). 2026 Sep 30;13:1916774. doi: 10.3389/fmed.2026.1916774 (PMC13661405; doi:10.3389/fmed.2026.1916774)
Supplement: Supplementary file 3 [file Table3.docx]

**Supplementary Table 3** Basic characteristics of the included literature(n=12)

| **Study (year)** | **Intervention** | **PAD Severity** | **Follow‑up** | **Multivariable Adjustment** |
| --- | --- | --- | --- | --- |
| Söderlund (2025) | Open, endovascular, or hybrid (mixed) | Claudication/CLTI | 5.9 years (median) | Yes |
| Morisaki (2020) | Revascularization (not specified) | CLTI | 2 years | Yes |
| Cao (2023) | Endovascular revascularization | CLTI | 3 years | Yes |
| Taniguchi (2019) | Open revascularization | CLTI | 3 years | Yes |
| Dağlı (2025) | Endovascular revascularization | PAD (unspecified) | 29.9±9 months (mean) | Yes |
| Matsubara (2017) | Revascularization (not specified) | CLTI | 3 years | Yes |
| Matsubara (2015) | Revascularization (not specified) | CLTI | 5 years | Yes |
| Selçuk (2023) | Surgical revascularization | CLTI | 30 days | Yes |
| Guler (2023) | Endovascular revascularization | CLTI | 1 year | Yes |
| Takino (2024) | Endovascular revascularization | PAD (unspecified) | 1 month | Yes |
| Sivaharan (2021) | Infrainguinal bypass | PAD (unspecified)/ CLTI | 21 months (median) | Yes |
| Shimazoe (2019) | Infrainguinal bypass | CLTI | 3 years | Yes |

Footnotes: PAD, peripheral artery disease; CLTI, chronic limb‑threatening ischemia
